# Prevention of cisplatin nephrotoxicity

**Published:** 2015-08-22

**Authors:** Fatemeh Hayati, Mehran Hossainzadeh, Shokouh Shayanpour, Zahra Abedi-Gheshlaghi, Seyed Seifollah Beladi Mousavi

**Affiliations:** ^1^Chronic Renal Failure Research Center, Ahvaz Jundishapur University of Medical Sciences, Ahvaz, Iran; ^2^Nickan Research Institute, Isfahan, Iran

**Keywords:** Cisplatin, Acute renal failure, Contrast-induced acute kidney injury

## Abstract

Cisplatin has a well-established role in the treatment of broad spectrum of malignancies; however its use is limited because of cisplatin-induced nephrotoxicity (CIN) which can be progressive in more than 50% of cases. The most important risk factors for CIN include higher doses of cisplatin, previous cisplatin chemotherapy, underlying kidney damage and concurrent treatment with other potential nephrotoxin agents, such as aminoglycosides, nonsteroidal anti-inflammatory agents, or iodinated contrast media. Different strategies have been offered to diminish or prevent nephrotoxicity of cisplatin. The standard approach for prevention of CIN is the administration of lower doses of cisplatin in combination with full intravenous hydration prior and after cisplatin administration. Cisplatin-induced oxidative stress in the kidney may be prevented by natural antioxidant compounds. The results of this review show that many strategies for prevention of CIN exist, however, attention to the administration of these agent for CIN is necessary.

**Implication for health policy/practice/research/medical education:**

Cisplatin is important chemotherapeutic agents used to treat solid tumors, including head, ovarian and neck, and testicular germ cell tumors, which its use is limited because of cisplatin-induced nephrotoxicity (CIN) that can be progressive in significant percent of patients. In the early trials prior to the use of preventive measures, the amount of acute renal failure (ARF) incidence resulting from this drug was observed in more than 5o% of cases. It is recommended that preventive measures must be used in all patients treated with cisplatin and aim of this mini-review is evaluation of these preventive measures.

## Introduction

Renal toxicity involves kidneys free radical production and increase, utilization of antioxidant defense mechanisms, and also acute renal tubular cells necrosis that leads to reduce glomerular filtration rate (GFR) and kidney dysfunction. Beside the pathological mechanisms involve rise of upregulation of transforming growth factor-β, endothelin-1, augmentation of oxidative stress, necrosis, apoptosis and increase in macrophage/monocyte infiltration into the renal cortex and medulla (1).

## Materials and Methods

For this review, we used a variety of sources by searching through PubMed, EMBASE, Scopus and directory of open access journals (DOAJ). The search was performed by using combinations of the following key words and or their equivalents; cisplatin, cisplatin-induced nephrotoxicity.

## Results

Cisplatin (cis-diamminedichloroplatinum II) is an antineoplastic agent drug, which is used in the treatment of a broad spectrum of malignancies including head and neck, esophagus, bladder, and metastatic testis, ovarian, breast and non-small cell lung cancer (1,2). Cisplatin is a strong cellular toxin and nephrotoxicity is one of the most important complication of this drug in clinical and experimental models, which can be progressive in more than 50% of cases (2). The highest concentration of cisplatin is observed in mitochondria, nuclei, cytosol and microsomes. Cisplatin is interlaced to glutathione and next metabolized through a gamma-glutamyl transpeptidase and a cysteine S-conjugate β-lyase-dependent pathways to a reactive thiol as a potent nephrotoxin (3). The kidney accumulates cisplatin by peritubular uptake and concentration of the drug achieved in the renal cortex is several folds greater than other organs. In addition more than 50% of the cisplatin is excreted in the urine in the first day following cisplatin administration (2). Acute renal failure (ARF) occurs due of damage to the kidney tissue caused by decreased renal blood flow or renal ischemia from any cause such as exposure to substances harmful to the kidney, low blood pressure and an inflammatory process in the kidney (1-3). Moreover, pathogenesis of acute kidney injury is complex. Also promoting events may be completely different, but similar pathways may be implicated in injury responses (3). Inhibition of pro-inflammatory mediators in cisplatin-induced ARF is associated with a decrease in renal neutrophils (4). Questions have been raised about the safety of prolonged use of cisplatin as a chemotherapeutic agent in the treatment of tumors.

## Antioxidant agents for prevention of cisplatin-induced nephrotoxicity

Generation of reactive oxygen species (ROS) and vasoconstriction in the renal microvasculature is responsible for the cisplatin-induced renal tubular injury. Moreover the S3 segment of the proximal tubule in the outer stripe of the outer medulla are selectively injured by cisplatin, as manifested by both apoptosis and necrosis. There have been a number of studies that interestingly reported, reactive nitrogen species (NOS) have also been involved in cisplatin-induced renal toxicity. Cisplatin intensifies the production of nitric oxide and peroxynitrite in renal tissues. Peroxynitrite stimulate changes in the function and structure of lipid peroxidation, chemical cleavage of DNA, proteins and reduction in cellular defenses by oxidation of thiol pools (3). Medicinal plants are a well source for variety of natural antioxidants and they are used for the treatment of diseases all over the world. Treatment with drugs and also drug discovery should be focused more than before on this natural source. Lately, researchers have shown an increased interest in finding natural antioxidants from plant sources for prevention and treatment of different diseases (5). Cisplatin-induced oxidative stress and inflammatory response in the kidney may partially be prevented by several chemical and natural compounds such as antioxidant. One study by Hemati et al, examined the effects of selenium and vitamin E on CIN in 22 patients, who received 200 μg selenium and 400 IU vitamin E daily and 24 patients received placebo. It has conclusively been shown differences in GFR between the two groups after the third cycle and also 1 month after the end of chemotherapy. They have been demonstrated that antioxidant agents including selenium and vitamin E and are effective in reducing oxidative toxicity of cisplatin (6). Many of studies suggested that ROS and mitochondrial damage involved in CIN (7,8). Delivery of antioxidants to mitochondria is one of the most important mechanism for prevention of CIN with the purpose of decreasing toxic oxidative stress injuries. Dimethylthiourea (DMTU) is a hydroxyl radical scavenger and demonstrated that all the following pathways in CIN, such as (*a*) increase plasmatic levels of blood urea nitrogen and creatinine, (*b*) decrease calcium uptake, ATP content and electrochemical potential, (*c*) depletion of the NADPH and glutathione (GSH) as an antioxidant defense, (*d*) oxidation of cardiolipin, sulfhydryl and aconitase enzyme, (*e*) increase activity of the apoptosis executioner including caspase-3 were prevented by DMTU (8). One study on rats showed that the combination of ebselen and allopurinol, which is a xanthine oxidase inhibitor with the potential to diminish ROS generation, reduced CIN. Ebselen as a glutathione peroxidase imitator, is a great scavenger of peroxynitrite (9).

Many of sulfur-containing compounds have been reduced the nephrotoxicity of cisplatin without inhibiting or decreasing its antitumor effect in patients with non-small cell lung cancer, metastatic breast cancer, ovarian cancer and metastatic colon cancer (3). In animal models and preliminary clinical studies, use of amifostine which is an organic thiophosphate, prior to cisplatin administration decreased incidence of CIN (10). According to these studies, some of authors suggested that amifostine could be considered in patients receiving repeated administrations of cisplatin for ovarian or non-small cell lung cancer and it limits toxicity by binding free radicals (11). A number of pharmacologic agents including N-acetylcysteine, theophylline and have also been offered to prevent and or diminish renal toxicity of this drug, however none of them has an established role (12,13). For example, in an animal study, theophylline which is a known nonselective adenosine receptor antagonist proposed for prevention of CIN (12). The result of this animal study is supported by clinical trial by Benoehr et al. In the study by Benoehr et al, prophylactic application of theophyline compared with placebo decreased the prevalence of CIN (14). However other additional clinical studies indicated that it has not a protective effect (13).

## Clinical manifestation and dosage of cisplatin

The most significant clinical manifestation of cisplatin nephrotoxicity is acute and chronic impairment of renal function, which are associated with major morbidity and mortality among these patients (15). Thrombotic microangiopathy, hypomagnesaemia, salt wasting, fanconi-like syndrome, and anemia are other clinical manifestation of cisplatin nephrotoxicity, which are occurred in significant percent of patients (16). The incidence of cisplatin nephrotoxicity and consequently clinical manifestation of that, have depended upon the dose and frequency of drug administration. There is relatively little information about risk factors for cisplatin nephrotoxicity. However it appears that the most important risk factors for cisplatin nephrotoxicity include higher doses of cisplatin that result in high peak plasma free platinum concentrations, previous cisplatin chemotherapy, inter-individual differences in cisplatin pharmacokinetics, patients with underlying kidney damage primarily due to the decreased renal clearance of cisplatin and concurrent treatment with other potential nephrotoxin agents such as aminoglycosides, non-steroidal anti-inflammatory drugs (NSAIDs), or iodinated contrast media (10,17).

The standard approach for prevention of CIN, which must be used in all patients treated with cisplatin is the administration of lower doses of that in combination with intravenous hydration prior and after chemotherapy administration. Although, the doses of cisplatin are not lowered in the treatment of malignancies such as small cell lung cancer and testicular germ cell tumors. The lower doses of cisplatin in conjunction with newer chemotherapy agents have been developed for palliative settings such as non-small cell lung cancer. Chemotherapy regimens with the lower doses of cisplatin have minimized the incidence of nephrotoxicity without lowering the therapeutic efficacy of cisplatin (17).

## Magnesium and CIN

Magnesium is one of the most common elements in the Earth. Magnesium is an important intracellular cation which is distributed into three major sections: intracellular space, extracellular fluid and mineral phase of bones. Magnesium-depletion is well-known side effects to cisplatin treatment. On the other hand, cisplatin induces magnesium depletion and magnesium deficiency itself may increase cisplatin nephrotoxicity (3). Lajer et al indicated a significant magnesium-depletion on CIN as evidenced by changes in renal failure induced mortality, plasma creatinine and urea and loss of renal transporters. On the other hand, magnesium-depletion enhances CIN (18). Magnesium supplementation is suggested to prevent the induced hypomagnesemia after cisplatin administration. In a non-randomized study by Beladi Mousavi et al, the patients were treated by cisplatin with equal dose or more than 50 mg/m^2^, received a solution consisting of 1000 ml of isotonic saline plus 20 mEq of potassium chloride (KCL) and 2 grams of magnesium sulfate (MgSo_4_)_._ They administered a minimum of 1000 ml of this solution over 2 to 3 hours prior and a minimum of 500 ml over the 2 hours after the cisplatin injection. The prescribed dose of the solution was to establish a urine flow of at least 100 cc/hour for two hours before and after chemotherapy. According to the results of this study, the amount of ARF incidence resulting from this drug in patients, who were hydrated with the mentioned protocol was 6.6% which is significantly less than the reported prevalence of this side-effect in patients, who are not hydrated (19). Hypokalemia and hypomagnesemia are well-known and frequent side effects of cisplatin, which occurs in more than half of patients receiving cisplatin-containing chemotherapy. It is also reported that hypomagnesemia may exacerbate cisplatin toxicity and cisplatin induced apoptosis. Therefore, it is suggested that in hydration protocol which are used for prevention of CIN, MgSo_4_ and KCl add to intravenous fluid for prevention of hypomagnesaemia, hypokalmia and their side effects (18,19).

## Prevention effect of saline and simultaneous

The accepted standard strategy is hydration with simultaneous and saline administration of mannitol after, during and before cisplatin administration that can significantly reduce CIN (3). Also demonstrated that saline alone or with furosemide has better renal protection in acute cisplatin nephrotoxicity than saline plus mannitol (20). Increase the rate of cisplatin excretion may attribute to volume expansion with hypertonic saline or saline. Moreover, salt provides a high concentration of chloride ions which can reduce the formation of the reactive, aquated species of cisplatin by preventing the separation of the chloride ions from the platinum molecule. According to an investigation by Hanigan et al, saline cannot alter the cellular accumulation of cisplatin but instead it release a stress response in the cell that modifies sensitivity to cisplatin (21).

## Conclusion

Cisplatin has a well-established role in the treatment of broad spectrum of malignancies; however its use is limited because of CIN which can be progressive in more than 50% of cases. The most important risk factors for CIN include higher doses of cisplatin, previous cisplatin chemotherapy, underlying kidney damage and concurrent treatment with other potential nephrotoxin agents, such as aminoglycosides, nonsteroidal anti-inflammatory agents, or iodinated contrast media. Different strategies have been offered to diminish or prevent nephrotoxicity of cisplatin. The standard approach for prevention of CIN is the administration of lower doses of cisplatin in combination with full intravenous hydration prior and after cisplatin administration. Cisplatin-induced oxidative stress in the kidney may be prevented by natural antioxidant compounds. The results of this review show that many strategies for prevention of CIN exist, however, attention to the administration of these agent for CIN is necessary.

## Authors’ contribution

FH, MH, SS and SSBM conducted the research. ZAG edited the paper. SSBM prepared the final manuscript.

## Conflicts of interest

The authors declared no competing interests.

## Ethical considerations

Ethical issues (including plagiarism, data fabrication, double publication) have been completely observed by the authors.

## Funding/Support

None.
